# Rocky Mountain Spotted Fever in a Large Metropolitan Center, Mexico–United States Border, 2009–2019

**DOI:** 10.3201/eid2706.191662

**Published:** 2021-06

**Authors:** Oscar E. Zazueta, Paige A. Armstrong, Adriana Márquez-Elguea, Néstor Saúl Hernández Milán, Amy E. Peterson, Diego F. Ovalle-Marroquín, Maria Fierro, Rodolfo Arroyo-Machado, Moises Rodriguez-Lomeli, Guillermo Trejo-Dozal, Christopher D. Paddock

**Affiliations:** Secretariat of Health of Baja California (Instituto de Servicios de Salud Pública del Estado de Baja California), Mexicali, Mexico (O.E. Zazueta, A. Márquez-Elguea, N.S.H. Milán, D.F. Ovalle-Marroquín, R. Arroyo-Machado, M. Rodriguez-Lomeli, G. Trejo-Dozal);; Centers for Disease Control and Prevention, Atlanta, Georgia, USA (P.A. Armstrong, A.E. Peterson, C.D. Paddock);; Imperial Country Public Health Department, El Centro, California, USA (M. Fierro)

**Keywords:** Rocky Mountain spotted fever, *Rhipicephalus sanguineus*, *Rickettsia rickettsii*, tickborne diseases, zoonoses, epidemiology, Mexico, Mexicali, bacteria, bacterial infections, rickettsial diseases, United States, vector-borne infections

## Abstract

Epidemic levels of Rocky Mountain spotted fe­­­ver (RMSF) have persisted in Mexicali, Mexico, since the initial outbreak was first reported in December 2008. We compared clinical and epidemiologic data of cases in Mexicali during 2009–2019 between patients with an IgG titer reactive with *Rickettsia rickettsii* bacteria by indirect immunofluorescence antibody (IFA) assay and those who demonstrated DNA of *R. rickettsii* in a whole blood sample when tested by PCR. We identified 4,290 patients with clinical and epidemiologic features compatible with RMSF; of these, 9.74% tested positive by IFA and 8.41% by PCR. Overall, 140 patients died (11-year case-fatality rate 17.97%). Substantial differences in the frequency of commonly recognized clinical characteristics of RMSF were identified between PCR-positive and IFA-positive cases. The Mexicali epidemic is unique in its size and urban centralization. Cases confirmed by PCR most accurately reflect the clinical profile of RMSF.

## Abstract 2

Longevity, high prevalence, and multifocal distribution of this disease pose unprecedented public health challenges.

Rocky Mountain spotted fever (RMSF), a severe and potentially deadly tickborne disease caused by *Rickettsia rickettsii* bacteria*,* occurs throughout the Americas. The classic epidemiology of RMSF is characterized by isolated and sporadic cases of disease that occur predominantly in rural or suburban settings (*1*). Occasionally, regional endemic foci of infection are described, which can persist for years, or sometimes decades (*2*). During the early 2000s, investigators identified multiple outbreaks of RMSF among several small communities in Arizona in the United States and in Sonora, Mexico (*3*–*6*). A feature common to each of these outbreaks has been the presence of large populations of stray and free-ranging dogs heavily infested with ticks. In these settings, canine populations can sustain and perpetuate massive numbers of brown dog ticks (*Rhipicephalus sanguineus* sensu lato), which serve as efficient vectors of *R. rickettsii* bacteria. 

In December 2008, cases of RMSF were first recognized among residents of a neighborhood in Mexicali, the capital city of Baja California, Mexico (*7*,*8*). During the next few years, cases were identified in adjacent and distant neighborhoods. In contrast to almost all previously described outbreaks of RMSF, this epidemic emerged within a large metropolitan center, continues in the present day, and has affected hundreds of persons throughout the city. Cases of RMSF are now also reported beyond the city limits from several small communities in the Mexicali Valley (*9*,*10*).

The ongoing epidemic of RMSF in Mexicali resembles past and present outbreaks in Arizona and northern Mexico. Cases of disease occur primarily in impoverished neighborhoods, where the presence of large populations of stray dogs infested with infected brown dog ticks greatly increase the human risk for exposure to the pathogen (*10*–*13*). Efforts to document the scope and magnitude of RMSF in Mexicali have been hampered by limited access to sensitive and specific diagnostic techniques, the relatively nonspecific clinical findings observed during the early stages of illness, and incomplete awareness among many residents and local health care providers of the regional risk and scope of the epidemic (*10*). To more accurately characterize the epidemiology of RMSF in Mexicali, we compiled and analyzed data available for all cases with serologic or molecular evidence of infection that were reported to the Secretariat of Health of Baja California (ISESALUD) during 2009–2019.

## Methods

### Setting

Mexicali is located at the Mexico–United States border, adjacent to the California town of Calexico. According to the National Institute of Statistics and Geography in Mexico, this large urban center extends across 114 km^2^ and has a population of ≈700,000 persons. The Mexicali Valley, comprising 13,700 km^2^, extends southeast of the city and is inhabited by ≈250,000 persons. Most city residents receive their medical care at hospitals and clinics operated by the Mexican Institute of Social Security (60%), ISESALUD (21%), and the Institute for Social Security and Services for State Workers (13%) (*14*).

### Collection of Data

We analyzed clinical and epidemiologic data for all cases of RMSF reported to ISESALUD in the Mexicali metropolitan area and the Mexicali Valley during 2009–2019 by abstracting data retrospectively from the standardized case report used by the Mexicali General Hospital and Ministry of Health clinics across the city (*15*) and all major hospitals in the area. Case definitions are established nationwide by the Directorate General of Epidemiology (DGE) (*15*,*16*). A probable case is defined as fever (>38.5°C) and >2 of the following signs or symptoms: headache, myalgia, rash, purpura, meningeal signs, alterations in cerebrospinal fluid, hemorrhage, liver enzyme abnormality, hematologic alterations, hyponatremia, leukocytosis, leukopenia, elevated levels of lactate dehydrogenase, or shock. Cases also require >1 of the following epidemiologic criteria during the 2 weeks preceding illness onset: history of tick bite or direct contact with a tick-infested dog, ticks identified in or around the patient’s household, or travel to or residence in a neighborhood where cases of RMSF had been recently identified. Confirmed cases include those in patients with a serum IgG titer >64 to *R. rickettsii* antigens, determined by an indirect immunofluorescence antibody (IFA) assay on serum samples, and those for whom PCR evaluation of a whole blood specimen demonstrates DNA of a spotted fever group *Rickettsia* (SFGR). For this investigation, we compared frequencies of clinical and epidemiologic characteristics identified for those patients with a positive PCR result with those identified by a positive IFA result.

### Laboratory Testing

During 2009, serum specimens were serially diluted beginning at 1:80; for all subsequent years, 1:64 was used as the first dilution. Antibody titers were expressed as the reciprocal of the last reactive dilution, and titers >64 were considered positive. Molecular analyses were performed by using a *Rickettsia* genus–specific real-time assay targeting a 74-bp segment of the citrate synthase gene and primers CS-F and CS-R and probe CS-P, as previously described (*17*). Samples with cycle threshold values <38 were considered positive on the basis of the cutoff for this assay established by the Mexico Secretariat of Health, Institute of Epidemiological Diagnosis and Reference.

### Mapping and Statistical Analyses

We transformed physical addresses of PCR-positive case-patients into geographic coordinates by using an automated algorithm developed by Mexico’s National Institute of Statistics and Geography. We created maps using Epi Info 7 (Centers for Disease Control and Prevention, https://www.cdc.gov/epiinfo). We described categorical variables as counts and proportions and continuous variables by using mean, median, and range. We used the *t*-test for comparisons of means and Fisher exact test for comparisons of proportions. Because of the large number of cases, we assumed that continuous data followed a normal distribution according to the central limit theorem. We did not report missing data. We used Stata 14 (StataCorp, https://www.stata.com) to perform all statistical analyses. This study was approved by the Institutional Review Board of Tijuana General Hospital (approval no. HGT-2017-000058).

## Results

**Figure 1:**
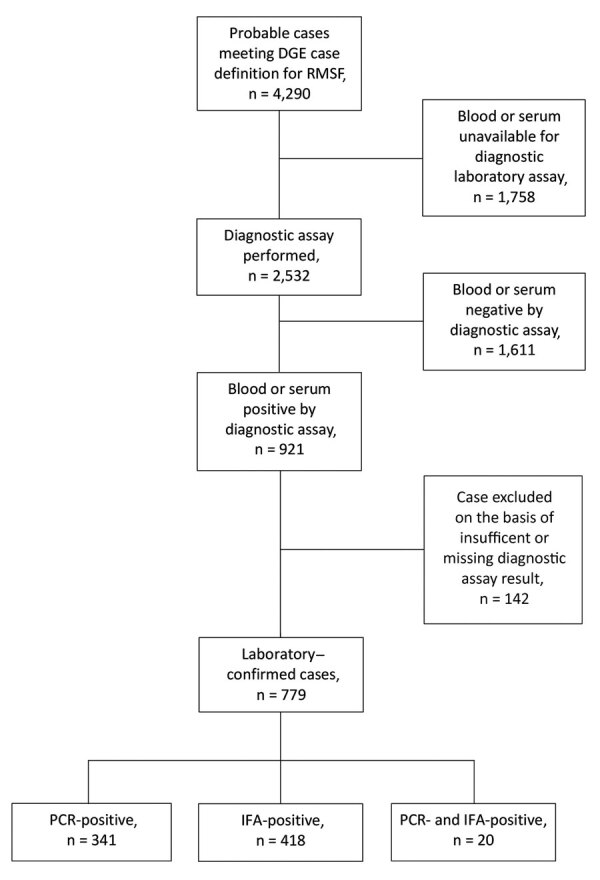
Flowchart used to determine case status of patients in whom Rocky Mountain spotted fever was diagnosed, Mexicali, Mexico, 2009–2019. DGE, Directorate General of Epidemiology; IFA, indirect immunofluorescence antibody assay; RMSF, Rocky Mountain spotted fever.

During 2009–2019, a total of 4,290 persons in metropolitan Mexicali and the Mexicali Valley had illnesses meeting the DGE case definition for probable cases of RMSF (Figure 1). Of those, a diagnostic assay was performed on 2,532 patients, and a positive result from either assay was recorded for 921 (36.37%). We excluded 142: those for whom only an IgM titer was available (n = 102) and those for whom an IFA result was reported as positive but without a reported antibody titer (n = 40). Of the total probable cases identified by the surveillance case definition, 779 (18.15%) met DGE criteria for a confirmed case, 418 (53.66%) with a positive test result by IFA and 361 (46.34%) by PCR (Table 1). For 341 confirmed cases, only PCR was performed, for 417 only IFA, and for 21 both tests. Of those tested by PCR and IFA, 20 had positive results with both assays, and 1 was negative by PCR but positive by IFA. 

**Table 1:** Frequency of laboratory-confirmed cases of Rocky Mountain spotted fever by assay, Mexicali, Mexico, 2009–2019*

Period	No. (%)			Total no. cases
	PCR-positive	IFA-positive	PCR- and IFA-positive	
2009–2011	73 (20.74)	275 (78.13)	4 (1.14)	352
2012–2015	172 (56.95)	114 (37.75)	16 (5.30)	302
2016–2019	96 (76.80)	29 (23.20)	0 (0)	125
Total	341 (43.77)	418 (53.66)	20 (2.57)	779

*IFA, indirect immunofluorescence antibody assay.

The median time from illness onset until the collection of whole blood for PCR and serum for IFA was 5 days (interquartile range [IQR] 3–8 days). The geometric mean titers of IFA-positive cases were 175 during 2009–2011, 231 during 2012–2015, and 156 during 2016–2019. Approximately two thirds (64.2%) of positive serum samples were collected during the first week of illness.

**Figure 2:**
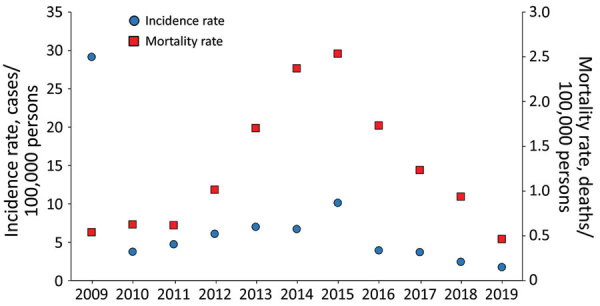
Incidence and mortality rates of laboratory-confirmed Rocky Mountain spotted fever, Mexicali, Mexico, 2009–2019. Scales for the *y*-axes differ substantially to underscore patterns but do not permit direct comparisons.

Nearly half (378, 48.52%) of all positive case-patients resided in the city of Mexicali. The remaining case-patients originated from several neighborhoods adjacent to but beyond the city limits (243 [31.19%]), the region of Puebla (57 [7.32%]), and from the Mexicali Valley (101 [12.97%]). The cumulative 11-year average incidence rate of RMSF during this period, including PCR- and IFA-confirmed cases, was 7.22/100,000 population/year (1.76–29.16/100,000 population/year) (Figure 2).

**Figure 3:**
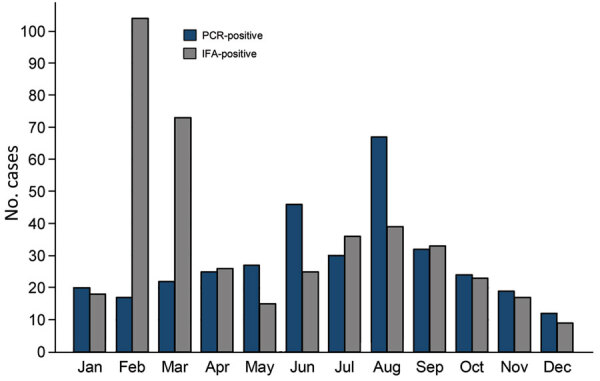
Seasonal distribution of PCR- and IFA-positive cases of Rocky Mountain spotted fever, Mexicali, Mexico, 2009–2019. IFA, indirect immunofluorescence antibody assay.

Among all confirmed case-patients, the mean age was 23.89 years (SD 17.65, IQR 9–36 years), which did not differ significantly between those whose cases were confirmed by IFA or PCR. Most patients (440 [56.48%]) were female. Cases occurred during each month of the year but were more frequent during the summer months (Figure 3). A disproportionately higher number of IFA-confirmed cases were identified during February and March; nonetheless, 156 (37.32%) of all IFA-positive cases were reported during early 2009, shortly after the outbreak was first recognized and before widespread access to confirmatory PCR assays. A total of 410 case-patients were hospitalized, 271 (66.1%) of whom were confirmed by PCR only, 123 (30%) by IFA only, and 16 (3.9%) by both assays. The mean length of stay of hospitalized case-patients was 8.89 days (median 5 days, IQR 1–11 days), a mean of 9.23 days for PCR-confirmed patients and 7.33 days for IFA-confirmed patients (p = 0.20).

Overall, 140 patients died (11-year case-fatality rate 17.97%): 125 (89.28%) whose illness was diagnosed by PCR only, 11 (7.86%) by IFA only, and 4 (2.86%) by both assays. Approximately one quarter of deaths occurred among children <15 years of age (Table 2). The case-fatality rate was 36.66% for PCR-positive and 2.63% for IFA-positive patients (p<0.001). PCR-confirmed case-patients were significantly more likely to be admitted to a hospital (p<0.001) and die from their infections than were case-patients confirmed by IFA (Table 3).

**Table 2:** Demographic and clinical characteristics of patients with PCR- and IFA-positive cases of Rocky Mountain spotted fever, Mexicali, Mexico, 2009–2019*

Characteristic	Total	PCR-positive	IFA-positive	p value
No. patients	759	341	418	
Sex				
F	433 (57.05)	170 (49.85)	263 (62.92)	<0.001
M	326 (42.95)	171 (50.15)	155 (37.08)	
Age, y (mean ± SD)	23.94 (± 17.67)	24.38 (± 18.89)	23.59 (± 16.62)	0.540
Hospitalized	394 (51.91)	271 (79.47)	123 (29.43)	<0.001
Died	136 (17.92)	125 (36.66)	11 (2.63)	<0.001
Signs and symptoms				
Fever	759 (100)	341 (100)	418 (100)	0.999
Headache	656 (86.43)	288 (84.46)	368 (88.04)	0.166
Myalgia	468 (61.66)	229 (67.16)	239 (57.18)	0.005
Arthralgia	403 (53.10)	197 (57.77)	206 (49.28)	0.023
Retro orbital pain	82 (10.80)	26 (7.04)	58 (13.88)	0.003
Rash	328 (43.27)	181 (53.24)	147 (35.17)	<0.001
Pruritis	139 (18.31)	56 (16.42)	83 (19.86)	0.258
Vomiting	322 (42.42)	188 (55.13)	134 (32.06)	<0.001
Nausea	366 (48.22)	206 (60.41)	160 (38.28)	<0.001
Chills	274 (36.10)	127 (37.24)	147 (35.17)	0.595
Photophobia	78 (10.28)	29 (8.50)	49 (11.72)	0.152
Abdominal pain	345 (45.45)	191 (56.01)	154 (36.84)	<0.001
Diarrhea	188 (24.77)	112 (32.84)	76 (18.18)	<0.001
Conjunctivitis	110 (14.49)	40 (11.73)	70 (16.75)	0.062
Nasal congestion	109 (14.36)	34 (9.97)	75 (17.94)	0.002
Cough	189 (24.93)	72 (21.18)	117 (27.99)	0.035
Pharyngitis	156 (20.58)	69 (20.23)	87 (20.86)	0.857
Rhinitis	106 (13.97)	34 (9.97)	72 (17.22)	0.004
Hepatomegaly	68 (8.96)	44 (12.90)	24 (5.74)	0.001
Splenomegaly	31 (4.08)	21 (6.16)	10 (2.39)	0.010
Adenomegaly	17 (2.24)	7 (2.05)	10 (2.39)	0.810
Jaundice	40 (5.27)	26 (7.62)	14 (3.35)	0.013
Hemorrhage	87 (11.46)	60 (17.60)	27 (6.46)	<0.001
Seizures	32 (4.22)	30 (8.80)	2 (0.48)	<0.001

*Values are no. (%) except as indicated. We excluded from these analyses 20 patients who were positive by both assays.

**Table 3:** Age distribution of patients with PCR- and IFA-positive cases of fatal Rocky Mountain spotted fever, Mexicali, Mexico, 2009–2019*

Age group, y	No. (%)			
	PCR-positive	IFA-positive	PCR- and IFA-positive	Total
<15	25 (20)	6 (54.55)	1 (25)	32 (22.86)
16–24	25 (20)	0	0	25 (17.86)
25–44	43 (34.40)	3 (27.27)	2 (50)	48 (34.29)
45–64	28 (22.40)	2 (18.18)	1 (25)	31 (22.14)
>65	4 (3.20)	0	0	4 (2.86)
Total	129 (100)	11 (100)	4 (100)	140 (100)

*IFA, indirect immunofluorescence antibody assay.

Among patients with laboratory-confirmed cases, the predominant signs and symptoms were fever (100%), headache (86.43%), myalgia (61.66%), arthralgia (53.10%), nausea (48.22%), abdominal pain (45.45%), and rash (43.27%). However, statistically significant differences were identified in the frequencies of several of these features, and many other clinical findings, when comparing PCR-positive versus IFA-positive patients (Table 2).

**Figure 4:**
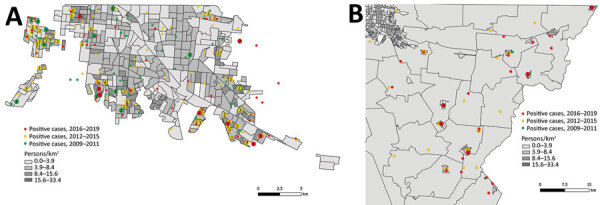
Geographic distribution of all PCR-positive cases of Rocky Mountain spotted fever in Mexicali (A) and the Mexicali Valley (B), Mexico, 2009–2019. Outlined areas represent census-related Basic Geostatistical areas established by Mexico’s National Institute of Statistics and Geography. Numbers in circles represent the number of cases in each location.

Geospatial analysis of PCR-confirmed cases during the periods 2009–2011, 2012–2015, and 2016–2019 revealed marked expansion of recognized cases across Mexicali. During the first 3 years of the outbreak, cases were concentrated predominantly in the western part of the city from where the index case originated in 2008. During 2012–2015, cases were subsequently identified in the southern portion of the city (Figure 4, panel A) and the region of Puebla in southeastern Mexicali. The Mexicali Valley also experienced progressively more cases during 2016–2019 (Figure 4, panel B). Cases have been identified in almost every neighborhood of Mexicali over the course of the epidemic, often repeatedly in the same areas over time.

## Discussion

The epidemiology of RMSF in Mexicali shares many features of epidemic RMSF identified previously among communities in other regions of southwestern North America (*3*–*6*,*13*). During the 1940s, investigators in Mexico were the first to identify and characterize the ecologic, epidemiologic, and social determinants of outbreaks of RMSF that emerged among small and impoverished communities of Sinaloa, Sonora, Durango, and Coahuila. These highly lethal outbreaks, ignited by unchecked canine populations that supported massive peridomestic infestations by brown dog ticks, resulted in high attack rates among women and children and ended in death for most infected patients (*13*,*18*,*19*). Contemporary outbreaks in Sonora and Coahuila also involve predominantly economically vulnerable populations and are particularly devastating among children, for whom mortality rates range from 30% to 57% (*13*,*20*–*22*). The Mexicali epidemic is similarly represented by a large number of pediatric patients and a nearly 37% case-fatality rate among PCR-positive cases. The year-round occurrence of the disease, with notable peaks during the summer and fall months, also reflects the previously described seasonal pattern of RMSF in northern Mexico.

The Mexicali epidemic is unique from all previously described outbreaks of RMSF in terms of its magnitude, urban concentration, and widespread persistence. The timing and origin of the introductory event that precipitated this multiyear outbreak remains unknown. However, the circumstances that propelled its expansion and eventual perpetuation across the city, including high-density, low-income neighborhoods with large numbers of free-roaming and stray dogs and abundant brown dog tick populations, exist within many other metropolitan areas across Mexico and Latin America. In this context, similar urban outbreaks could plausibly originate elsewhere after local introduction of *R. rickettsii.* As we note, during 2009–2019, surveillance activities by ISESALUD identified 779 patients with laboratory-supported diagnoses of RMSF in Mexicali and the Mexicali Valley. By comparison, the largest modern outbreak of RMSF in the United States, involving 466 confirmed and probable cases during 2003–2019, has affected predominantly rural tribal communities in Arizona (https://www.azdhs.gov/preparedness/epidemiology-disease-control/index.php#data-stats-past-years). Urban foci of RMSF are described only rarely and sporadically in the United States and other countries of Latin America and are characteristically limited in size and duration (*23*–*26*), so the longevity, remarkably high prevalence, and multifocal distribution of RMSF in a large metropolitan center poses unprecedented public health challenges.

This study is also noteworthy for the large number of PCR-positive cases available for analysis. Various studies indicate that the clinical sensitivities of molecular assays are low early in the illness, but increase as the disease progresses and the patient becomes severely ill (*27*,*28*). PCR-positive patients are more likely to have severe manifestations, which could bias comparisons between groups confirmed by molecular and serologic methods. In addition, the molecular assay used to confirm cases of RMSF in Mexicali is specific only for the genus *Rickettsia* (*17*); because other pathogenic *Rickettsia* species, including *R. massiliae*, *R. parkeri*, and *R. typhi*, are endemic to northern Mexico (*29*–*33*), some PCR-positive patients identified in this series might have represented cases of rickettsial diseases other than RMSF. However, the overall severity of illnesses, coupled with extensive and consistent epidemiologic and environmental evidence implicating brown dog ticks as the principal vector perpetuating this outbreak (*8*–*10*,*12*), suggest strongly that most, if not all, PCR-confirmed cases were indeed infections caused by *R. rickettsii*.

Although IFA methods are used widely for epidemiologic evaluations of RMSF, the use of a single IgG titer can reflect past exposure to an SFGR at an undetermined time and can inaccurately reflect surveillance estimates that define the magnitude and clinical characteristics of RMSF (*29*). Because IgG titers are reflective of the host immune response to *R. rickettsii*, these titers are not expected to be elevated in the first several days of illness, when most patients seek medical attention. In fact, ≈50% of patients with RMSF lack a diagnostically relevant IFA titer (i.e., >64) during the first week of illness (*34*). In addition, >50% of all deaths attributed to *R. rickettsii* occur within 7–9 days after illness, which explains the large percentage of persons who die from RMSF without serologic confirmation (*35*). In this investigation, approximately two thirds of the IFA-confirmed case-patients for whom an illness onset date was recorded had a titer at or above the threshold value for a positive result during the first week of illness. For these reasons, we compared PCR-positive cases to those with only a positive IFA result and identified substantial differences between these groups. The clinical characteristics of PCR-positive case-patients in Mexicali matched closely with those described for well-characterized series from Arizona, USA, and Coahuila and Sonora, Mexico (*3*,*22*,*36*,*37*). In contrast, case-patients with a single IgG titer were less likely to demonstrate many of the classical characteristics of RMSF, including rash, myalgia, abdominal pain, nausea, and vomiting. In addition, the frequencies of hospitalization, jaundice, hemorrhages, seizures, and death were each less common for IFA-positive case-patients. These findings indicate that some or many cases defined by a single positive antibody titer do not accurately reflect the clinical profile of RMSF in Mexico, and that subsequent case definitions for RMSF should require confirmation by molecular methods specific for *R. rickettsii*, or a >4-fold rise in IgG titers between paired serum samples.

Because IgG titers reactive with *R. rickettsii* can persist in some persons for >1 year after resolution of the acute infection, some, or perhaps many, of the IFA-positive cases could represent patients exposed to or infected remotely with an SFGR who sought treatment for other febrile, rash-associated diseases endemic to northern Mexico, including dengue, Zika virus infection, or leptospirosis (*13*). Furthermore, we excluded from our analyses ≈1,600 probable case-patients for whom laboratory tests were negative; nonetheless, some, or perhaps many, of these probable cases reflected actual cases of RMSF, particularly those in patients tested early in the course of disease and for whom PCR or IFA methods were unable to detect rickettsial DNA or antibodies reactive with *R. rickettsii*. Collectively, these situations could skew frequencies of clinical characteristics and case-fatality rates, pose limitations to the tabulation of actual cases, and preclude accurate assessment of the incidence of RMSF in Mexicali during the period of study.

During 2009–2019, several hundred foci of RMSF emerged and recurred within multiple neighborhoods across the city of Mexicali, suggesting a transition from epidemic to hyperendemic disease. Similarly concerning is the more recent recognition of RMSF among smaller rural communities of the Mexicali Valley. The longevity and multifocal distribution of RMSF in Mexicali underscore many of the complex challenges faced by public health authorities in this expansive urban setting. Achieving a level of acceptable risk will require coordinated and sustained control and prevention strategies that diminish substantially the numbers of *R. rickettsii*–infected *Rh. sanguineus* s.l. ticks and free-roaming and stray dogs across a densely populated region covering >100 km^2^. Because brown dog ticks are predominantly endophilic and spend ≈95% of their life hidden in structural cracks and crevices of human habitations and surrounding structures, this species can be notoriously difficult to control. Surreptitious infestations and high fecundity rates can result in explosive increases in *Rh. sanguineus* s.l. tick populations. Acaricides that contain pyrethoids, including permethrin and cypermethrin, are commonly used in these peridomestic settings because of their relative safety to nontarget species; nonetheless, resistance to these compounds has been identified recently among some brown dog tick populations in Mexico (*38*).

The ecology of RMSF in Mexicali, as in other regions of the southwestern North America with hyperendemic or epidemic levels of disease, is linked inextricably to an overabundance of tick-infested free-ranging and stray dogs in affected communities (*3*,*4*,*10*–*13*,*19*,*39*). Results of a citywide canine serosurvey conducted in Mexicali in 2017 identified antibodies reactive to *R. rickettsii* in 65% of 213 owned dogs, and 55% of all examined animals were infested with brown dog ticks (*10*). Levels of tick infestation and exposure to *R. rickettsii* are likely even greater among stray dogs (*30*,*39*). Movement of free-ranging, tick-infested canines within and among neighborhoods could contribute to multifocal recurrences of RMSF identified in Mexicali during the 11-year study period. Community-based interventions that provide and apply long-acting, acaracide-impregnated collars to large numbers of dogs can bring about rapid and substantial declines in canine and environmental tick populations and cases of RMSF among community inhabitants (*10*,*40*). Nonetheless, the distribution of stray dogs in cities correlates closely with high-density, low-income areas, and sustained interventions are prohibitively expensive for most affected neighborhoods (*40*). In this context, funding from state, national, or international agencies is needed to establish and maintain collaring activities, animal control, and spay and neuter programs that reduce the amplification of brown dog ticks and *R. rickettsii*.

In conclusion, intensified clinical and public education on the regional ecology of RMSF in Mexicali, and the necessity for rapid diagnosis and appropriate treatment, are of paramount importance as the outbreak in this location continues. The emergence and perpetuation of RMSF in Mexicali and in several other states of northern Mexico are not isolated or anomalous outbreaks but rather should be considered harbingers of national or even international concern (*41*). The enormous human and economic costs associated with epidemic RMSF will undoubtedly continue without adoption and use of well-supported and carefully integrated efforts that directly address this public health emergency.
